# Diphtheria

**Published:** 1906-03-24

**Authors:** 


### DIPHTHERIA.

An important contribution to the practical study of diphtheria is to be found in the current number of "Public Health," from the pen of Mr. H. J. Hutchens. Firstly, as regards the identification of the bacillus of diphtheria. This is often a matter of great difficulty, but the points upon which reliance is to be placed are: (*a*) the appearance of polar staining seen when Neisser's stain (*i.e.*, an acid solution of methylene blue) is used with Bismarck-brown as a counter-stain. With these stains the bodies of the bacilli appear brown, but the granules, which are usually polar, present a deep blue colour. (*b*) The *Bacillus diphtheriæ* when grown in glucose broth produce an acid reaction of the medium in forty-eight hours. This acid-producing capacity is a peculiarity of special importance. (*c*) Animal experiment. An injection of 1 c.c. of a forty-eight-hour broth culture of the diphtheria bacillus will kill a guinea-pig in about forty-eight hours, the post-mortem appearances consisting of a local œdema, reddening of the suprarenal bodies, and a pleural effusion. (*d*) Immunity; by repeated inoculation of sub-lethal doses of the bacilli or toxins a susceptible animal may be rendered immune to the diphtheria bacillus.

Several varieties of organism are liable to be confounded with the diphtheria bacillus. Some of these, which the author calls "diphtheroid," are indistinguishable from the true diphtheria bacillus except by the circumstance that they are not pathogenic to laboratory animals. For practical purposes these have still to be included among the diphtheria bacilli, of which some authorities consider them to be an attenuated form. The organism most likely to be confounded with the diphtheria bacillus is that described by Löffler and Hofman, and commonly called the "pseudo-diphtheria" or "Hofman's" bacillus. This organism is also considered by some observers to be a modified form of the diphtheria bacillus, but the author is convinced that the two are totally distinct. Extensive evidence is given to support the differentiation, in particular the facts that Hofman's bacillus never yields typical polar staining, is never pathogenic to laboratory animals, and does not produce an acid reaction in glucose broth. Further, Petrie asserts "that no substances capable of neutralising diphtheria antitoxin are present in the filtrates of the pseudo-diphtheria bacillus," and that "the results of the immunisation of horses with large quantities of the filtrates of the pseudo-diphtheria bacilli make it apparent that they do not contain substances capable of stimulating the production of an antitoxin to diphtheria toxin." Hofman's bacillus is, in the opinion of the author, a normal inhabitant of the mouth, and occurs more frequently in the mouths of the classes who consistently neglect the care of the teeth than among those who have been taught the elements of personal hygiene. The diphtheria bacillus, on the other hand, although it may be recovered from apparently healthy throats, is not found except in the throats of those who have been exposed to infected people—that is to say, the diphtheria bacillus is not one of the normal flora of the mouth.

The origin of an outbreak of diphtheria is often very difficult to trace, because infected "contacts" who themselves give no clinical evidence of the disease are perfectly competent to disseminate the infection, as, of course, are mild cases which escape notice or are wrongly diagnosed, and convalescents who are discharged before their throats are bacteriologically clean. Some excellent examples are given of the means by which outbreaks are initiated. In one case a pupil at a girls' private boarding-school contracted diphtheria. As soon as the disease was recognised the throats of all the inmates of the establishment were examined and diphtheria bacilli detected in the throat of the cook alone. On being questioned, she admitted having been to a neighbouring town some three or four days previously. While there she had been in contact with a case of diphtheria. The cook herself was in perfect health. In another instance a child with nasal diphtheria had infected six out of eight members of his class, in addition to his father, mother, and all his brothers and sisters, before he was found out. The mother said that the child had had a "stuffy cold in the head" for some three weeks or more. This one unsuspected case was responsible for no less than sixty-four cases of clinical diphtheria. Epidemics, further, are occasionally due to the infection of milk, in which medium the bacillus of the disease grows readily. An excellent example, somewhat too long for reproduction here, emphasises the importance of this avenue. Toys alone, such as india-rubber or other dolls, are very liable to hand on the infection from hand to mouth. Bad drainage and other un-hygienic conditions of life can only be regarded, in the light of bacteriological researches, as contributory causes, active on account of the lowered state of health resulting from them. As regards treatment, the first essential is the early recognition of the disease in order to secure the early administration of the antitoxin. The antitoxin can only antagonise the poison of the bacillus before it has undergone chemical union with the tissues, and therefore postponement of the administration is fatal to success.

Mr. Hutchens deals at some length with the question of prophylaxis and the dosage of antitoxin, but space does not permit of an adequate allusion to some of the matters of importance touched upon under these headings. In general terms the paper under notice is a valuable exposition of the current attitude of the best opinion in the matter of diphtheria.

